# Cellular Cytoskeleton Dynamics Modulates Non-Viral Gene Delivery through RhoGTPases

**DOI:** 10.1371/journal.pone.0035046

**Published:** 2012-04-11

**Authors:** Anandika Dhaliwal, Maricela Maldonado, Clayton Lin, Tatiana Segura

**Affiliations:** 1 Biomedical Engineering Interdepartmental Program, University of California Los Angeles, Los Angeles, California, United States of America; 2 Chemical and Biomolecular Engineering Department, University of California Los Angeles, Los Angeles, California, United States of America; University of Birmingham, United Kingdom

## Abstract

Although it is well accepted that the constituents of the cellular microenvironment modulate a myriad of cellular processes, including cell morphology, cytoskeletal dynamics and uptake pathways, the underlying mechanism of how these pathways influence non-viral gene transfer have not been studied. Transgene expression is increased on fibronectin (Fn) coated surfaces as a consequence of increased proliferation, cell spreading and active engagement of clathrin endocytosis pathway. RhoGTPases mediate the crosstalk between the cell and Fn, and regulate cellular processes involving filamentous actin, in-response to cellular interaction with Fn. Here the role of RhoGTPases specifically Rho, Rac and Cdc42 in modulation of non-viral gene transfer in mouse mesenchymal stem (mMSCs) plated in a fibronectin microenvironment was studied. More than 90% decrease in transgene expression was observed after inactivation of RhoGTPases using difficile toxin B (TcdB) and C3 transferase. Expression of dominant negative RhoA (RhoAT19N), Rac1(Rac1T17N) and Cdc42 (Cdc42T17N) also significantly reduced polyplex uptake and transgene expression. Interactions of cells with Fn lead to activation of RhoGTPases. However, further activation of RhoA, Rac1 and Cdc42 by expression of constitutively active genes (RhoAQ63L, Rac1Q61L and Cdc42Q61L) did not further enhance transgene expression in mMSCs, when plated on Fn. In contrast, activation of RhoA, Rac1 and Cdc42 by expression of constitutively active genes for cells plated on collagen I, which by itself did not increase RhoGTPase activation, resulted in enhanced transgene expression. Our study shows that RhoGTPases regulate internalization and effective intracellular processing of polyplexes that results in efficient gene transfer.

## Introduction

Although gene delivery can be a robust approach to treat disease and augment tissue formation, limitations with effective and save delivery have limited its success as a therapy. Gene delivery to mammalian cells can be achieved using viral as well as non-viral delivery systems [1]. Non-viral gene delivery systems have the advantage of being less immunogenic compared to viral gene delivery systems as well as allow for large-scale production and modularity. However, they are limited by their efficacy. Previous studies have focused on engineering more efficient delivery vehicles that can overcome one or more of the barriers to efficient gene transfer [2], [3]. Although less common, recent studies have looked at the cellular microenvironment and the cell itself to elucidate other approaches to enhance non-viral gene transfer [4]–[8]. For example, the stiffness of the matrix where the cells are plated modulates non-viral gene delivery with cells plated on stiff surfaces (110 KPa) resulting in enhanced gene transfer due to increased cell proliferation and survival [8]. Collagen I and IV have been shown to enhance gene transfer in PC12 cells, which was correlated with the relative projected nuclear area of the plated cells [9], while fibronectin and collagen I have been shown to enhance gene expression in NIH/3T3 cells which has been correlated to the relative increased internalization on these surfaces and the pathway of internalization [11]. Cationic lipid-mediated gene transfer to rat smooth muscle cells is enhanced when the cells are plated on surfaces that promote α_v_ß_3_ binding, with antibodies against α_v_ß_3_ and ß_3_ decreasing the amount of gene transfer [9]. Further, our laboratory has shown that cellular microenvironment modulates non-viral gene delivery to mouse mesenchymal stem cells (mMSCs), specifically we screened 6 different ECM proteins and their combinations for their ability to enhance gene transfer to mouse mesenchymal stem cells (mMSCs) using poly(ethylene imine) polyplexes. We found that proteins that promoted well spread cells (e.g. fibronectin and collagen IV) resulted in polyplexes being trafficked to the nucleus and enhanced gene transfer, while those that resulted in less spread cells (e.g. collagen I) resulted in polyplexes that did not colocalize with the nucleus and inhibited gene transfer [10]. When comparing the internalization pathway of polyplexes for cells seeded on fibronectin or collagen I, we found that different endocytic pathways are used, with clathrin-mediated endocytosis being the primary pathway used for cells plated on fibronectin [6]. Further, polymerized actin, actin-myosin interactions, and the microtubular network were found to influence non-viral gene transfer to different extends for cells seeded on fibronectin versus collagen I [6].

Structural components of the ECM such as Fn are able to actively mediate crosstalk between the ECM and RhoGTPases by associating with cell surface receptors, namely integrins [11] and syndecans [12], which effectively engage RhoGTPases leading to adhesion signaling [13], [14]. Rho proteins alternate between an active GTP-bound state and an inactive GDP-bound state. In the active conformation, GTPases interact with and stimulate the activity of effectors which participate in signaling cascades that coordinate various cellular processes such as migration, proliferation [15], gene expression [16] and cytoskeletal organization. Studies have shown that Cdc42 mediates cell polarity and filipodia, Rac mediates protrusion of lamellipodia, and Rho maintains cell adhesion during migration [17]. Furthermore, bacterial internalization [18], [19], adenovirus internalization [20] and recently receptor mediated internalization of transferrin [21] has been shown to be a resultant of host cell actin cytoskeleton manipulation at level of RhoGTPases. However, the role of RhoGTPases in non-viral gene transfer has not been previously investigated. It is likely that the interplay between Fn and integrins is communicated via the RhoGTPases [22] and the resultant signaling cascades affecting cytoskeletal dynamics, endocytosis, gene transcription and proliferation are regulating non-viral gene transfer in cells plated on Fn. Herein, to begin to understand the cellular molecules involved in modulating gene delivery to mMSCs plated on Fn coated surfaces, the role of Rho, Rac and Cdc42 RhoGTPases in the uptake and transgene expression of DNA/PEI polyplexes was studied. The RhoGTPases were specifically inhibited using chemical inhibitors and dominant negative gene products, as well as activated to determine their role in gene transfer and DNA/PEI internalization.

## Results

### Fibronectin exposure activates RhoGTPases in mMSCs

**Figure 1:**
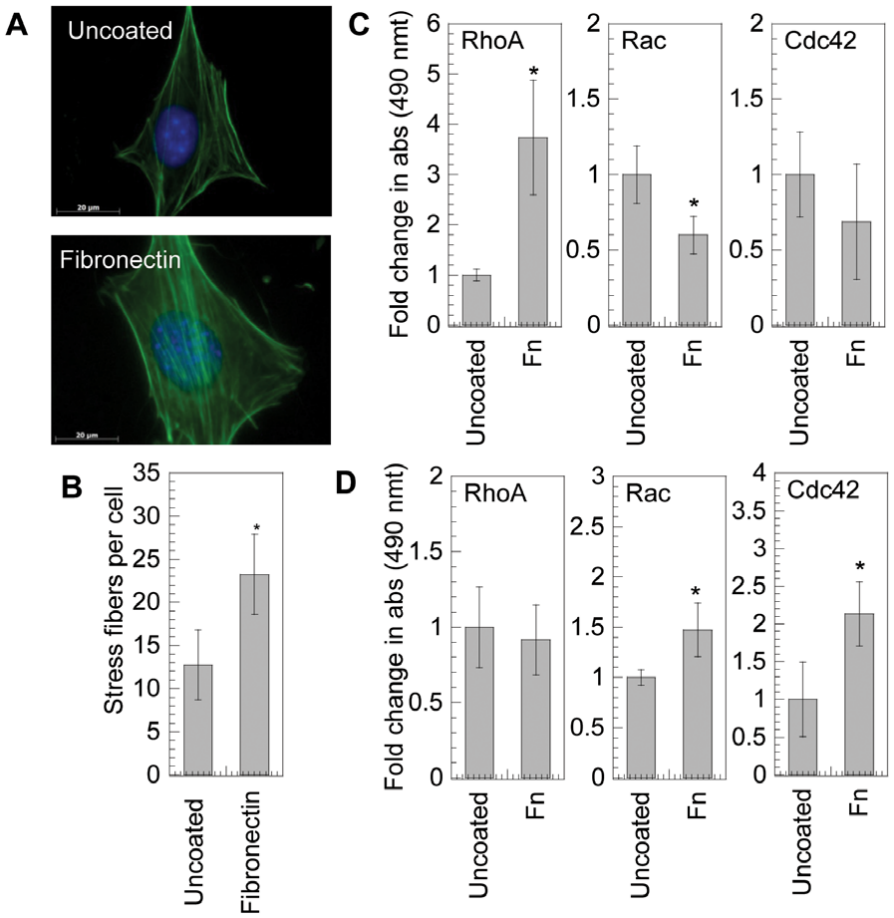
RhoGTPase activation on fibronectin. (**A**) mMSCs were plated on uncoated or fibronectin (40 µg/mL) coated surfaces for 16 hours and cell morphology was studied by staining cells for actin using Alexa488 conjugated phalloidin (green), and for DNA using Hoechst 33258 dye (blue). Images were taken with a Zeiss AxioObserver Z1 inverted microscope at 100× magnification. (**B**) Stress fiber quantification. (**C and D**) Active RhoGTPase quantification was performed using GLISA specific for RhoA, Rac1,2,3 and Cdc42. Cells were culture in PDMS sheets and allowed to attach and spread before exposing them to protein modified plastic for 20 minutes or 1 hour. The average of three samples was plotted. The level of active RhoGTPase in samples exposed to fibronectin was compared with untreated sample using the unpaired t-test (two tail p value). The symbols * represents a significant change to the level of p<0.05. The error bars represent the standard deviation in all plots.

The activation of RhoGTPases in mMSCs by fibronectin was assed using fluorescence immunohistochemistry and molecular assays. Fluorescence immunohistochemistry of the actin cytoskeleton revealed that cells plated on fibronectin-coated surfaces resulted in an increase in stress fibers compared to cells plated on tissue culture plastic (Fig. 1A, B, p<0.05), indicative of active RhoGTPases. The level of RhoGTPase activation was determined using GLISA assays for RhoA, Rac1,2,3 and Cdc42. To ensure that the observed activation was due to the interaction of the cell with the fibronectin coated surface and not the dynamics of cell spreading, cells were allowed to attach and spread on amine functionalized PDMS sheets. Following cell spreading, the cell sheet was flipped on top of fibronectin coated or uncoated tissue culture plastic and incubated for 20 minutes or 1-hour. At 20 minutes of exposure to fibronectin-coated surfaces, RhoA was significantly activated (p<0.05), while Cdc42 was not activated and Rac1,2,3 was inhibited compared to exposure to bare tissue culture plastic (Fig. 1C). After 1-hour exposure to fibronectin-coated surfaces, Rac1,2,3 and Cdc42 were significantly activated (p<0.05), while RhoA was not activated (Fig. 1D).

### Inactivation of RhoGTPases for cells plated on fibronectin inhibits transgene expression

**Figure 2:**
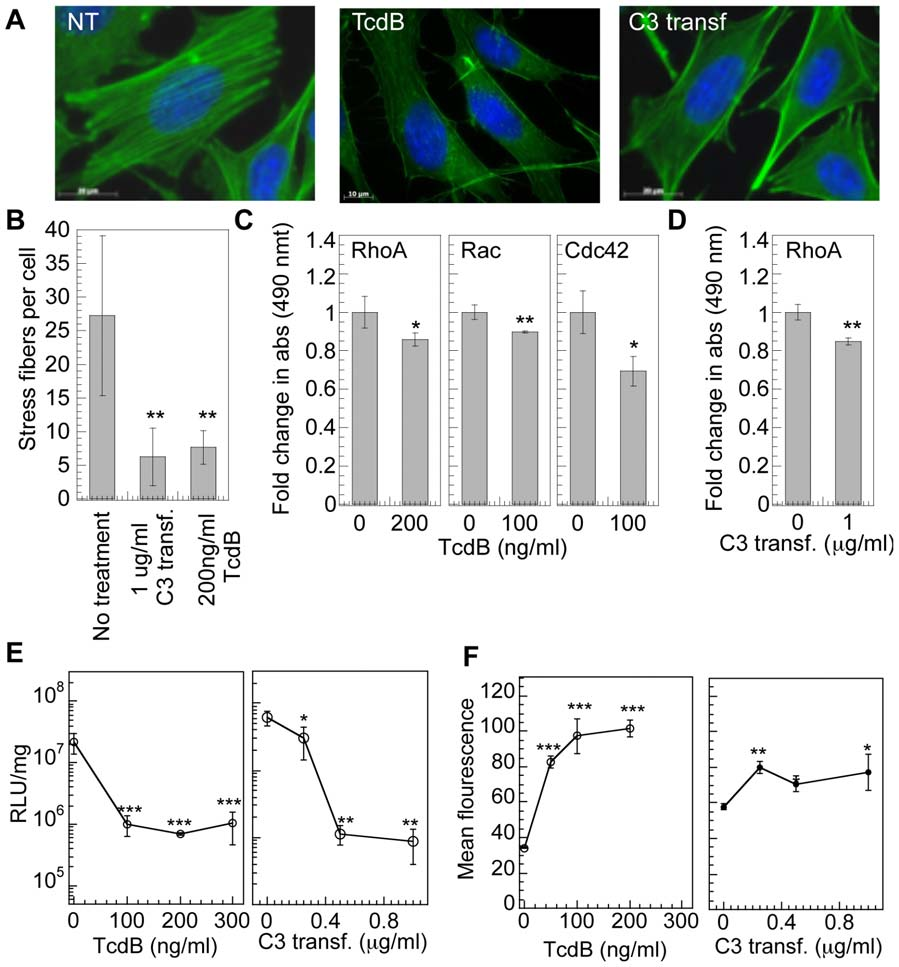
RhoGTPase inhibition using TcdB and C3 transferase decreases transgene expression. mMSCs were plated on fibronectin (40 µg/mL) coated tissue culture plastic for 16 hours prior to being treated with 0–300 ng/ml TcdB for 4 hours in serum free media, or 0–1 µg/ml C3 transferase for 4 hours. (**A**) Cell morphology was studied immediately after treatment with inhibitor, by staining cells for actin using Alexa488 (green) conjugated phalloidin, and for DNA using Hoechst 33258 dye (blue). Images were taken with a Zeiss AxioObserver Z1 inverted microscope at 100× magnification. (**B**) Stress fiber quantification. Statistical analysis was done using a one-way Anova followed by the Dunnett Multiple Comparison test. The Anova p value was 0.0052. The symbol ** represents a significant change in stress fibers with respect to untreated cells to the level of p<0.01. (**C**) Active RhoGTPase quantification was performed using GLISA specific for RhoA, Rac1,2,3 and Cdc42. (**D**) Active RhoA quantification was performed using GLISA specific for RhoA. Statistical analysis was done using the unpaired t-test (two tail p value) where active RhoGTPase level in treated cells was compared with untreated cells. The symbols * and ** represents a significant change to the level of p<0.05 and p<0.01, respectively. (**E**) Cells were transfected immediately post treatment and transgene expression was analyzed at 48 hours using luciferase assay and normalized with total protein quantified using Peirce BCA assay. (**F**) Cells were transfected immediately post treatment with YOYO-1 labeled polyplexes and after 2 hours cells were collected with trypsin and polyplex internalization analyzed by flow cytometry. A total of 7000 events were analyzed per sample. Experiments were performed in triplicate and the average plotted with the error bars representing the standard deviation. Fold increase in transgene expression and internalization was calculated with respect to the untreated control. Statistical analysis for gene transfer and internalization was done using a one-way Anova followed by the Tukey-Kramer Multiple Comparison test, which compares all columns. The Anova p values for transgene expression were 0.004 and 0.0052 for TcdB and C3 transferase, respectively and the Anova p values for internalization were <0.0001 and 0.0067 for TcdB and C3 transferase, respectively. The symbol ***, ** and * represents a significant change in gene expression or internalization with respect to sample not treated with inhibitor to the level of p<0.001, p<0.01 and p<0.05, respectively.

Inactivation of RhoGTPases was used to screen their potential involvement in mediating non-viral gene transfer in mMSCs. Inactivation of RhoGTPase using difficile toxin B was characterized using actin staining and GLISAs for RhoGTPases. Cells with inactivated RhoGTPases resulted in less spread cells with a significant decrease in stress fibers (p<0.01, Fig. 2A, B) and amount active RhoA, Rac1,2,3 and Cdc42 (p at least<0.05, Fig. 2C). Cell viability was not significantly affected immediately after treatment with TcdB and 48 hrs post transfection (Fig. S1A). RhoA,B,C were selectively inactivated using C3 transferase and resulted in a significant decrease in actin stress fibers (p<0.01, Fig. 2A, B) and a significant decrease in active RhoA (p<0.01, Fig. 2D). Cell viability was maintained at >60% immediately post C3 transferase treatment as well as 48 hours after treatment and transfection (Fig. S1B).

Cells were transfected immediately following the inhibitory treatments and transgene expression was analyzed using a luciferase assay at 48 hours and the extent of polyplex internalization was assessed using flow cytometry analysis (FACS) after 2-hours. More than 90% decrease in transgene expression of cells plated on fibronectin-coated surfaces was observed after treatment with TcdB and C3 transferase (p<0.001, Fig. 2E). However, internalization of the complexes was significantly increased following treatment (p at least<0.05, Fig. 2F).

**Figure 3:**
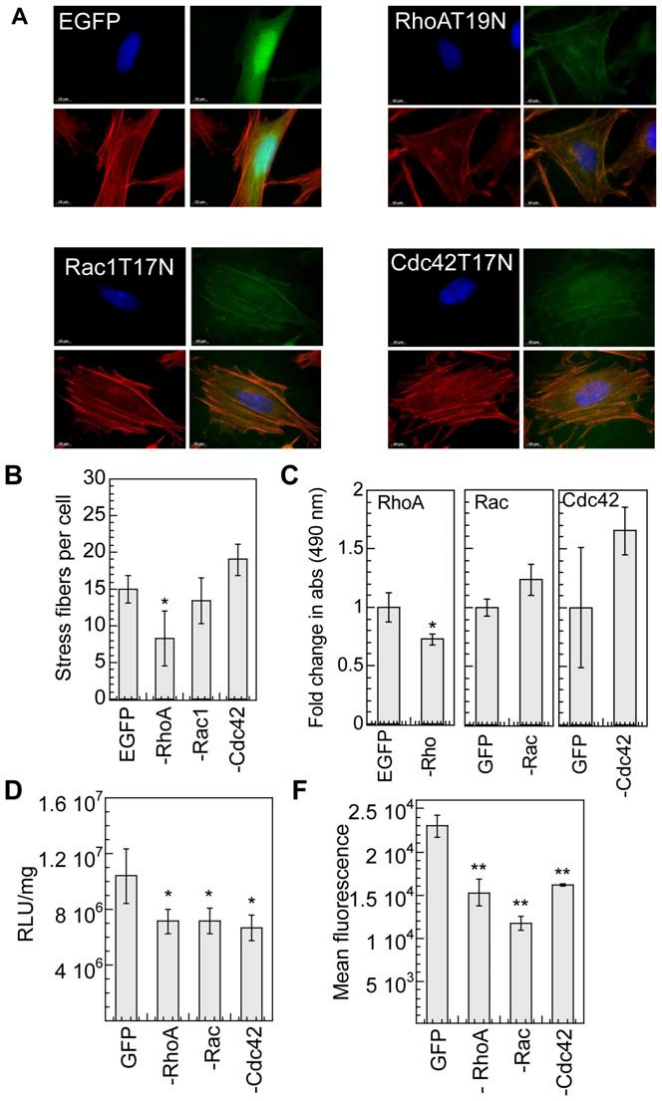
RhoGTPase inhibition using dominat negative gene products decreases transgene expression. mMSCs were transiently transfected with dominant negative forms of RhoA, Rac1 or Cdc42 with a plasmid also containing EGFP. 24 hours post transfection, cells were replated on fibronectin coated plates and cultured for 16 hours prior to bolus transfection. Cells transfected with pEGFP were used as control. (**A**) Alexa488 conjugated phalloidin (green), and Hoechst dye (blue) staining. Images were taken with a Zeiss AxioObserver Z1 inverted microscope at 100× magnification. (**B**) Stress fiber quantification. Statistical analysis was done using a one-way Anova followed by the Dunnett Multiple Comparison test. The Anova p value was 0.0015. The symbol * represents a significant change in stress fibers with respect to untreated cells to the level of p<0.05. (**C**) Active Rho, Rac and Cdc42, was assessed for cells replated on fibronectin for 16 hours using GLISA assays specific for RhoA, Rac1,2,3 and Cdc42. Statistical analysis was done using the unpaired t-test (two tail p value). The symbol * represents a significant change to the level of p<0.05. (**D**) The transgene expression was analyzed 48 hours post transfection using luciferase assay and normalized with total protein analyzed using Peirce BCA assay. (**E**) Internalization was analyzed using YOYO-3 labeled polyplexes 2 hours post transfection using flow cytometry. A total of 10,000 events were analyzed per sample and the mean fluorescence of events positive for both GFP and YOYO-3 was analyzed. Statistical analysis for gene transfer and internalization was done using a one-way Anova followed by the Dunnett Multiple Comparison test. The Anova p values were 0.0219 and <0.0001 for transfection (D) and internalization (E) respectively. The symbols * and ** represents a significant change with respect to cells transfected with control plasmid (pEGFP) to the level of p<0.05 and p<0.01, respectively.

To further understand the individual role of RhoGTPases, mMSCs were transiently transfected with GFP fused plasmid constructs of dominant negative mutants of either RhoA (RhoA-T19N), or Rac1 (Rac1-T17N) or Cdc42 (Cdc42-T17N) [19], [23]. At least 80% cell viability was maintained in cells expressing EGFP control plasmid and dominant negative genes 16-hours post plating cells on fibronectin (at time of bolus transfection) and 48 hrs post gene delivery (Fig. S2C). Expression of RhoA-T19N by cells plated on fibronectin coated surface resulted in a significant decrease (p<0.01) in stress fibers; however, no significant difference in stress fibers was observed in cells expressing dominant negative mutants of Rac1 and Cdc42 (Fig. 3A, B). GLISA analysis of active Rho, Rac1,2,3 and Cdc42 showed a significant decrease in active RhoA upon transfection with RhoA-T19N (p<0.05), but no change in active Rac or Cdc42 upon transfection with Rac1-T17N or Cdc42-T17N compared to cells transfected with EGFP (Fig. 3C).

Transgene expression was significantly reduced in the cells expressing dominant negative form of RhoA, Rac1 and Cdc42 (p<0.05, Fig. 3D). The effect of dominant negative mutants on internalization was studied by analyzing the uptake of YOYO-3 labeled complexes in GFP positive cells only. A significant decrease in internalization was observed for cells expressing dominant negative form of RhoGTPases (p<0.01, Fig. 3E).

### Activation of RhoGTPases for cells plated on fibronectin does not influence transgene expression

**Figure 4:**
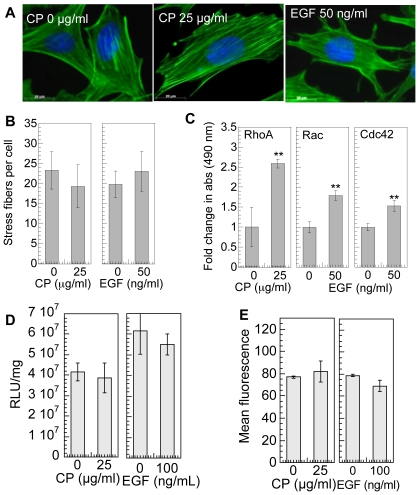
Effect of RhoGTPase activation using calpeptin (CP) and epidermal growth factor (EGF) on gene transfer. mMSCs cells were cultured for 8 hours on fibronectin wells, followed by overnight serum starvation and then treated with 0–100 µg/ml CP for 10 minutes or 0–100 ng/ml EGF for 2 minutes in serum free media. (**A**) After treatment, the changes in cell morphology were visualized through lexa488 conjugated phalloidin (green) and for DNA using Hoechst 33258 dye (blue) staining. Images were taken with a Zeiss AxioObserver Z1 inverted microscope at 100× magnification. (**B**) Stress fiber quantification. (**C**) Active RhoGTPase quantification after treatment was performed using GLISA specific for RhoA, Rac1,2,3 and Cdc42. (**D**) Transfection was performed immediately post treatment in serum free media. 4 hours post transfection media was replaced with complete media. The transgene expression was analyzed 48 hours post transfection using luciferase assay and normalized with total protein analyzed using Peirce BCA assay. (**E**) Internalization was analyzed 2 hours post transfection using flow cytometry of YOYO-1 labeled polyplexes. A total of 7000 events were analyzed per sample. Statistical analysis for the level of active RhoGTPase, transgene expression and internalization, was done using the unpaired t-test (two tail p value) where treated sample was compared with untreated sample. The symbols ** and *** represents a significant change to the level of p<0.01 and p<0.001, respectively.

Since RhoGTPase inactivation lead to a significant decrease in gene transfer efficiency for cells plated on fibronectin-coated surfaces, next we wanted to determine if further activation of RhoGTPases enhanced transgene expression. Calpeptin (CP) was used to activate RhoA [24], [25]. Treatment with CP did not significantly change the number of stress fibers in the cell (Fig. 4 A, B), but did significantly increased the amount of active Rho (by 2.5-fold) (p<0.01, Fig. 4C) compared to cells seeded on fibronectin coated surfaces that did not receive treatment. Viability was maintained at 90% or greater compared to cells not treated with CP immediately after treatment as well as 48 hours post treatment and addition of polyplexes (Fig. S1C). Epidermal growth factor (EGF) is an efficient activator of Rac1,2, and Cdc42 [26]. Treatment with EGF resulted in a significant increase in active Rac1,2,3 and Cdc42 (p<0.01 and <0.01, Fig. 4C) compared to cells seeded on fibronectin-coated surfaces that did not receive treatment. No significant change in cell viability was observed immediately post treatment and 48 hours post treatment and transfection (Fig. S1D). No significant difference in transgene expression (Fig. 4E) and internalization (Fig. 4F) was observed using treatment with CP or EGF for cells placed on fibronectin.

**Figure 5:**
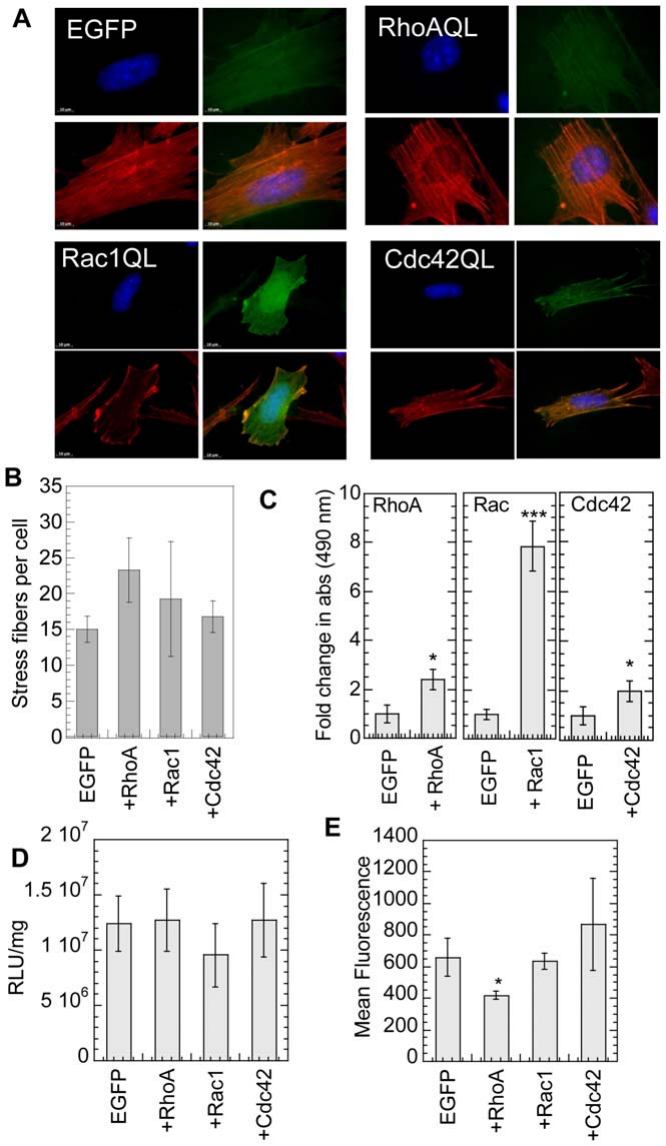
Effect of expression of constitutively active mutants of RhoGTPases on gene transfer. mMSCs plated on a 6-well plate were transiently transfected with constitutive active forms of RhoA, Rac1 or Cdc42 conjugated with GFP, using lipofectamine™2000. Cells transfected with pEGFP were used as control. After 24 hours, cells were harvested and replated on fibronectin-coated surfaces. (**A**) 24 hours post transfection with constitutive active forms of RhoGTPases in cells plated on tissue culture plastic, changes in cell morphology were analyzed through actin and DNA staining using Alexa488 conjugated phalloidin (green), and Hoechst 33258 dye (blue), respectively. (**B**) The number of stress fibers per cell were counted. (**C**) Active Rho, Rac and Cdc42-GTPases were assessed using GLISA assays 24 hours post transfection with constitutive active forms of RhoGTPases and overnight serum starvation. (**D**) The replated cells were cultured for 16 hours on fibronectin before bolus transfection. The transgene expression was analyzed 48 hours post transfection using luciferase assay and normalized with total protein analyzed using Peirce BCA assay. (**E**) Internalization was analyzed 2 hours post transfection using flow cytometry and YOYO-3 labeled polyplexes. A total of 10,000 events were analyzed per sample and the mean fluorescence of events positive for both GFP and YOYO-3 was analyzed. Statistical analysis for number of stress fibers, ruffles, transgene expression and internalization was done using the Dunnett multiple comparison test.

To confirm the CP and EGF findings, mMSCs were transiently transfected with GFP fused plasmid constructs of constitutive active mutants of either RhoA (RhoA-Q63L), or Rac1 (Rac1-Q61L) or Cdc42 (Cdc42-Q61L) [23]. At least 80% cell viability was maintained in cells expressing EGFP control plasmid and active genes 16 hours post plating cells on fibronectin (at time of bolus transfection) and 48 hours post gene delivery (Fig. S2D). Transfection with constitutively active RhoA mutant resulted in an increase in stress fiber formation (Fig. 5A, B). Further, a significant increase in the amount of active RhoA, Rac1 and Cdc42 was observed for cells transfected with the constitutively active genes (p at least<0.05, Fig. 5D). Similar to the results obtained for CP and EGF further activation of RhoGTPases did not enhance transgene expression further over that observed for cells seeded on fibronectin and transfected with EGFP (Fig. 5D). Expression of constitutively active form of RhoA, Rac1 and Cdc42 did not affect internalization (Fig. 5E). These results confirm that further activation of RhoA, Rac1 or Cdc42 over what fibronectin achieves on its own (Fig. 1 C, D) does not alter transgene expression.

### Activation of RhoGTPases for cells plated on collagen I enhances transgene expression

**Figure 6:**
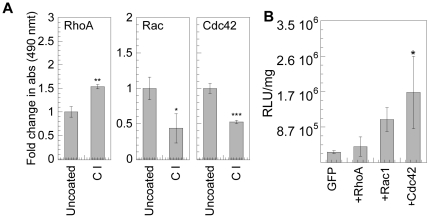
Effect of expression of constitutively active mutants of RhoGTPases on gene transfer for cells cultured on collagen I substrates. (**A**) mMSCs were cultured on amine functionalized PDMS strips and serum starved overnight before exposing to collagen I coated surfaces for 20 minutes to assess Rho-GTP and 1 hour to assess Rac and Cdc42-GTPases. The cells were subsequently lysed and respective GLISA assays were performed to quantify RhoA, Rac1,2,3 and Cdc42. Statistical analysis was done using the unpaired t-test (two tail p value). (**B**) mMSCs transiently transfected with constitutively active negative of RhoA, Rac1 or Cdc42 conjugated with GFP were cultured on collagen I coated plates for 16 hours prior to bolus transfection with polyplexes. The transgene expression was analyzed 48 hours post transfection using luciferase assay and normalized with total protein analyzed using Peirce BCA assay. Statistical analysis was done using a one-way Anova followed by the Dunnett Multiple Comparison test. The Anova p value was 0.0203. The symbols *, ** and *** represents a significant change to the level of p<0.05, p<0.01 and p<0.001, respectively.

Activation of RhoGTPases for cells plated on collagen I was significantly lower compared to cells plated on fibronectin (Fig. 6 vs. Fig. 1). Thus, we used transfection experiments on collagen I to further test our hypothesis of the importance of active RhoGTPases on non-viral gene transfer. Rho was enhanced 4-fold on fibronectin coated surfaces but only 1.5-fold for cells plated on collagen I. Rac was enhanced 1.5-fold for cells plated on fibronectin but inhibited by 50% for cells plated on collagen I. Cdc42 was enhanced by 2-fold for cells plated on fibronectin, but inhibited by 50% for cells seeded on collagen I (Fig. 6A). Cells were transfected with constitutively active RhoA, Rac1 and Cdc42 and replated on collagen I coated surfaces for 16 hours before transfection. Rac1 and Cdc42 activation were able to enhance the transgene expression by 4.1 and 6.7 fold compared to EGFP mock transfected cells respectively, while RhoA activation had no difference (Fig. 6B). Since RhoA was activated by cell exposure to collagen I (Fig. 6A), this result shows that further activation of RhoA is not beneficial to achieve higher transgene expression similarly to what was observed for cells plated on fibronectin.

### Rho effector protein Rho-Kinase (ROCK) and PKC mediated phosphorylation modulate gene transfer

**Figure 7:**
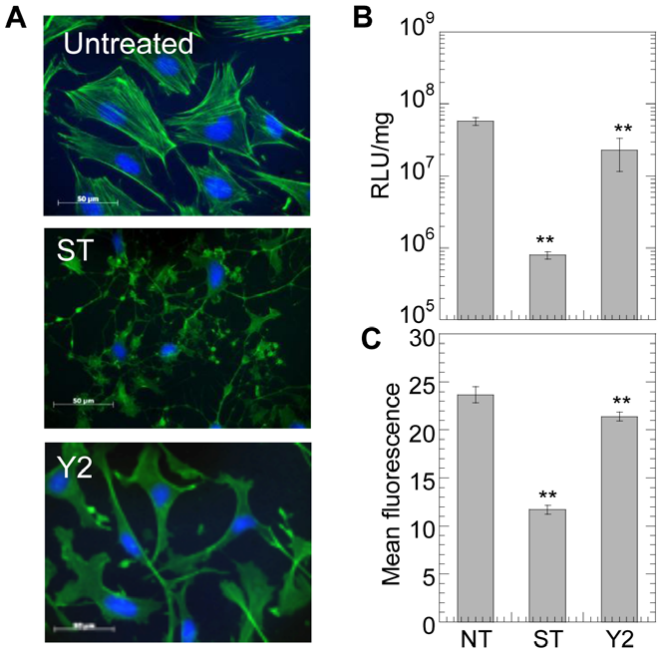
PKC and ROCK inhibition, inhibit gene transfer in cells plated on fibronectin. mMSCs were cultured for 16 hours on fibronectin coated plates prior to treatment with 10 µM Y27632 (Y2) or 100 nM Staurosporine (ST) for 2 hours to inhibit ROCK and PKC, respectively. (**A**) Changes in mMSC cell morphology were visualized through staining for actin using Alexa488 conjugated phalloidin and for DNA using Hoechst 33258 dye. Images were taken with a Zeiss AxioObserver Z1 inverted microscope at 40× magnification. Immediately after treatment with inhibitor, the medium was replaced and bolus transfection was done. (**B**) Transgene expression was analyzed 48 hours post transfection using luciferase assay and normalized with total protein analyzed using Peirce BCA assay. Internalization was assessed 2 hours post transfection using flow cytometry and YOYO-1 labeled polyplexes (**C**). A total of 7000 events were analyzed per sample. Statistical analysis was done using a one-way Anova followed by the Dunnett Multiple Comparison test. The Anova p value was 0.0003 and <0.0001 for transgene expression and internalization, respectively. The symbols ** represents a significant change to the level of p<0.01.

ROCK was inhibited using Y27632 (Y2) and PKC was inhibited using staurosporine (ST). Treatment with Y27632 reduced the number of actin stress fibers while treatment with staurosporine lead to a loss of cystoskeletal integrity (Fig. 7A). No significant change in cell viability was observed immediately after inhibitor treatment. However, 48 hours post treatment and transfection, ST significantly reduced (p<0.05) cell viability while Y2 did not affect the cell viability (Fig. S1E). Y27632 is a specific inhibitor of ROCK. Treatment with Y27632 resulted in a significant decrease in overall transgene expression and polyplex internalization (p<0.01, Figs. 7B and C), further suggesting the importance of Rho in non-viral gene transfer. PKC inhibition resulted in a severe decrease in transgene expression and polyplex uptake (P<0.01).

## Discussion

The process of non-viral gene delivery involves various steps, including cellular internalization, trafficking along the endo-lysolytic path, endosomal escape, unpackaging, nuclear entry and gene expression [27]. Modulation of any of these steps can influence the final transgene expression. While recent studies show that cellular microenvironment regulates gene delivery [4], [7], the mechanisms activated upon ECM binding that influence effective gene transfer have not been elucidated. Previously we found that mMSCs plated on fibronectin coated surfaces resulted in enhanced gene transfer, while cells plated on collagen I resulted in inhibited gene transfer compared to cells seeded on uncoated surfaces [6]. Here we aimed to determine if RhoGTPase activation was one of the reasons for the observed enhancement in transgene expression for cells plated on fibronectin-coated surfaces.

RhoGTPases have been primarily implicated as the controlling factors in guiding cell motility, morphology, contractility and proliferation [17]. RhoGTPases have also been implicated in regulating phagocytosis [28], endocytic trafficking [29], as well as mediating the various steps of vesicular trafficking [30]. Thus, RhoGTPases can play a role in polyplex uptake and polyplex intracellular trafficking. Fibronectin has been previously shown to enhance signaling through RhoGTPases [11], [14], [31], [32]. Initial activation of Rac and Cdc42 by fibronectin has been shown to mediate cell spreading in NIH/3T3 cells [14], [32]. Our results show that exposure of mMSCs to fibronectin coated surfaces results in enhanced activation of the RhoGTPases namely RhoA, Rac1,2,2 and Cdc42, in mMSCs. To study the role of RhoGTPases on non-viral gene transfer for mMSCs plated on fibronectin coated surfaces, systematic inhibition and activation by molecular inhibitors as well as dominant negative or positive genes were used.

RhoGTPases were inhibited using bacterial toxins (TcdB and C3 transferase) and dominant negative genes. TcdB irreversibly inactivates Rho, Rac and Cdc42 by glycosylation of the threonine residue [33], [34], while C3 selectively inhibits RhoA, B and C [35], but not Rac or Cdc42, by modifying the aspargine41 at the effector region of the GTPase. Inhibition by bacterial toxins resulted in a significant decrease (at least p<0.05) in the overall amount of active Rho, Rac and Cdc42. Further, a significant decrease (p<0.05) was observed in active RhoA upon transfection with RhoAT19N but no change was observed in active Rac or Cdc42 upon transfection with Rac1T17N and Cdc42T17N, respectively. Since for both inhibition protocols cells are seeded on fibronectin, these results indicate that the dominant negative gene could inhibit RhoA even though the cells are plated on fibronectin, but the dominant negative gene could only reduce the activation of Rac and Cdc42 to background levels when the cells are plated on fibronectin.

Consistent with our hypothesis of the involvement of RhoGTPases on non-viral gene transfer, inhibition of RhoGTPases led to a significant decrease in transgene expression. Although the exact mechanism of how RhoGTPases influence successful non-viral gene transfer cannot be determined from our data, we predict that the regulation of actin-microtubule dynamics, cell proliferation [15] and gene transcription via integrin-dependent intracellular signaling pathways [16] positively influence non-viral gene transfer. RhoGTPases have been implicated in endocytosis of bacteria and viruses by mammalian cells. For example, Rac1, RhoA, and Cdc42 are required for HeLa cell invasion by group B streptococcus [19], RhoA positively regulates the pathway by which Madin-Darby canine kidney (MDCK) cells internalize P. aeruginosa strain PA103 [18], and Rho C enhances macropinocytosis and modulates entry of Ebola virus and vesicular stomatitis virus pseudotyped vectors.

Interestingly the effect on polyplex internalization was not the same for the bacterial toxins and transfection with dominant negative genes. While inhibition of RhoGTPases using TcdB and C3 transferase led to an increase in polyplex internalization, inhibition with dominant negative genes led to a decrease in internalization. We cannot offer a definitive reason for these findings. The observed increase in internalization on treatment with TcdB could be due to TcdB mediated activation of phospholipase C and D [34], leading to regulation of receptor mediated endocytosis [36], [37]. The increase observed on C3 treatment correlates with our previous study where depolymerization of actin using cytochalasin D enhanced polyplex uptake in mMSCs cultured on fibronectin [6]. The observed decrease in internalization for inhibition with dominant negative genes could possibly be due to inhibition of macropinocytosis by dominant negative mutants of Cdc42 and Rac1, as was shown for bone marrow derived dendritic cells [38].

Since we found that exposure of mMSCs to fibronectin activates RhoGTPases (1.5- to 4-fold) and enhances transgene expression, we wanted to determine if further activation of RhoGTPases could further enhance transgene expression. RhoGTPases were activated using calpeptin, EGF and transfection with constitutively active RhoGTPase mutants. Calpeptin (CP) is a cell permeable calpain inhibitor known to activate Rho leading to stress fiber formation [24], [25]. Treatment with calpeptin and EGF did not influence the transgene expression or polyplex uptake. Although we show that EGF activates Rac and Cdc42, EGF also stimulates macropinocytosis [39]. Therefore, the role of activated RhoA, Rac1 and Cdc42 was specifically studied by transiently expressing constitutively active RhoGTPase mutants. Transfection with constitutively active RhoGTPases resulted in increased amounts of active RhoA, Rac and Cdc42. However, like CP and EGF treatment, the expression of constitutively active RhoA, Rac1 and Cdc42 did not influence transgene expression or uptake for mMSCs plated on fibronectin. These results indicate that a further activation of RhoGTPases does not increase transgene expression in mMSCs cultured on fibronectin-coated dishes.

As mentioned we previously observed that gene transfer to mMSCs plated on collagen I was inhibited [6]. Thus, we investigated if the activation of RhoGTPases could rescue the transfection ability of mMSCs plated on collagen I. For mMSCs exposed to collagen I coated dishes, only Rho was slightly activated, while both Rac and cdc42 were inhibited. The activation of Rac1 and Cdc42 with constitutively active genes increased the transgene expression by 4.1 and 6.7 fold, respectively, for cells plated on collagen I coated surfaces. As was the case for cells seeded on fibronectin, the further activation of RhoA by transfection with a constitutively active RhoA gene does not further stimulate gene transfer. On the other hand, Rac and Cdc42 were inhibited on Collagen I coated surfaces, and thus the fact that transfection with constitutively active Cdc42 and Rac1 increased transgene expression indicates that these RhoGTPases can promote transgene expression in cells with less active RhoGTPases.

The small RhoGTPases employ downstream effectors to mediate their effects on cell morphology, focal adhesion and migration. RhoGTPase binds to serine/threonine kinases [40] namely Rho Kinase, ROK and the related p160ROCK; and elevate their activity. The role of Rho downstream effector ROCK in non-viral gene transfer was also investigated. ROCK inhibition using Y27632 significantly decreased the polyplex internalization and transgene expression. This suggests that cell contractility mediated by ROCK contributes to the regulation of polyplex uptake by RhoGTPase. Besides ROCK, there are other Rho effector molecules like Phosphatidyl inositol 4-phosphate 5-kinase (PIP5-kinase) and p140mDia that also regulate actin polymerization [41], [42] and could play a role in mediating gene transfer and would be interesting to study in future.

Besides integrins, syndecans also mediate cell-Fn interactions [43] and activate PKC, which acts upstream to activate RhoGTPases [44] and mediate cell contractility. Syndecan-4 directly recruits PKC-α and facilitates focal adhesion formation on fibronectin [45]. To determine the function of PKC in non-viral gene transfer, it was inhibited using staurosporine. A decrease in transgene expression was seen on PKC inhibition as a resultant of decreased internalization and proliferation. PKC stimulation has been shown to induce clathrin mediated endocytosis of α_5_β_1_ [46] and actin dependent uptake of cationic DNA complexes [47]. These results indicate RhoGTPase activity regulated by PKC [44] is required for effective transgene expression.

In conclusion, our results indicate that RhoGTPases modulate non-viral gene transfer for mMSCs plated on fibronectin-coated surfaces. Our results show that the engagement of the ECM dictates the RhoGTPase activation level, which in turn dictates the efficiency of transgene expression. Activation of RhoGTPases for cells with inactive RhoGTPases leads to enhanced transgene expression, but activation of RhoGTPases for cells with already active RhoGTPases does not result in further enhancement. Since only RhoA, Rac1 and Cdc42 were studied we cannot offer conclusions about the involvement of RhoGTPases (e.g. RhoB/C)nin non-viral gene transfer. We believe that a molecular understanding of the process of non-viral gene transfer is necessary to engineer systems that achieve efficient gene delivery.

## Materials and Methods

### Cell Culture

Mouse bone marrow cloned mesenchymal stem (D1, CRL12424) were purchased from ATCC (Manassas, VA, USA). Cells were maintained in Dulbecco's modified eagle's medium (Sigma-Aldrich, St. Louis, MO) containing 10% bovine growth serum (BGS, Hyclone, Logan, Utah) and 1% penicillin/streptomycin antibiotics (Invitrogen, Grand Island, NY) and cultured at 37°C and 5% CO_2_.

### Protein coating

Stock solution fibronectin (1 mg/ml, Millipore, Billerica, MA) was diluted to 40 µg/ml in phosphate buffered saline (PBS), and added in each well of a tissue culture plate. The plate was incubated over night at 4°C in a humid environment, followed by incubation at 37°C for 2 hours [48]. The solution was removed and wells were washed twice with PBS to remove unbound proteins. Wells were further incubated with BSA (1% in PBS) for 30 minutes at 37°C followed by two washings with PBS.

### Analysis of RhoGTPase activation by fibronectin or collagen I

Poly-dimethoxy silane (PDMS) sheets were made by mixing Sylgard 184 PDMS prepolymer thoroughly in a 10∶1 mass ratio of silicone elastomer to curing agent, degassing and casting in a square dish at 60°C overnight. After the cured PDMS sheets were removed, they were cut into strips and oxygen plasma treated using Technics RIE (Reactive Ion Etching) for 3 minutes at 100 mTorr of O2, 100 W or 15 minutes at 200 mTorr of O2, 200 W. The PDMS strips were subsequently placed in a 1∶1 v/v methanol and hydrochloric acid solution for 30 minutes, then dried and incubated overnight in a 5% v/v amino-propyl triethoxysilane (APTES, Sigma-Aldrich, St. Louis, MO) solution in ethanol, at room temperature and in inert atmosphere [49]. The sheets were sterilized in 70% ethanol prior to cell plating. The cells were cultured for a day till confluent and then serum starved overnight before exposing them to fibronectin or collagen I coated surfaces. Five minutes before exposing cells to fibronectin or collagen I, sodium orthovanadate (0.1 mM) was added to the cell culture media. Sodium orthovanadate is an inhibitor of protein tyrosine phosphatases and alkaline phosphatases and was used to preserve the phosphorylation of RhoGTPases through inhibiting endogenous phosphatases [14].

The PDMS cell sheets were exposed to fibronectin or collagen I for 20 min or 1 hour, which have been shown to be the duration of RhoGTPase activation in different cells after interaction with the extracellular matrix [11], [14], [31], [32]. After exposure to ECM protein at 37°C, the strips were placed in Petri dishes on ice and rinsed once with ice cold PBS supplemented with 0.2 mM sodium orthovanadate. The lysis buffer provided with the GLISA kit (Cytoskeleton, Inc., Denver, CO) was modified to include 5 mM sodium orthovanadate, 50 mM sodium fluoride and 0.5 mM phenylmethylsulfonyl fluoride (PMSF), along with 1× protease inhibitor cocktail provided with Kit [14], [50] After aspirating all liquid from the PDMS strips, 100 ul of lysis buffer was added and scraped. Cell lysate was immediately clarified by centrifugation at 9,000 rcf, 4°C for 2 minutes, aliquoted into microcentrifuge tubes and snap frozen in liquid nitrogen, as indicated by the manufacture's protocol. After 1 or 2 days, the frozen aliquots were analyzed and RhoGTPases activity assessed, as per manufacture's protocol.

### Chemical inhibition of RhoGTPases

Difficile toxin B (TcdB, List Biologicals, Campbell, CA) was used to pharmacologically inhibit Rho, Rac and Cdc42 [18]–[20], [28], [51]. Rho was specifically inhibited using cell permeable C3 transferase (C3, Cytoskeleton, Denver, CO) [18], [28], [51]. mMSCs were seeded on fibronectin coated 48-well plates or 24-well plates at cell densities of 20,000 and 50,000 cells/well respectively. The cells were allowed to attach for 14–15 hours before the media in the wells was replaced with serum free media containing 0–1 µg/ml C3 transferase (C3) or 0–300 ng/ml TcdB for 4 hours. Immediately post treatment, media was changed with serum containing media and DNA/PEI internalization and overall transgene expression studied. To assess RhoGTPase inhibition, cell morphology was analyzed and respective GLISA assays were performed, immediately after inhibitor treatment.

### Chemical activation of RhoGTPase

Cells were allowed to attach on fibronectin-coated plates for 8 hours followed by overnight incubation in serum free media. Rho was pharmacologically activated by s incubating cells in presence 25 µg/ml Calpeptin (CP, Cytoskeleton, Inc., Denver, CO) for 10 minutes while Rac and CDC42 were activated by incubating cells in presence of 50 ng/ml Epidermal growth factor (EGF, Cytoskeleton) for 2 minutes. Immediately after treatment cell morphology, internalization and gene expression were analyzed. For analysis of non-viral gene transfer, cells were transfected with polyplexes in serum free media for 2–4 hrs, following which media was replaced with serum containing media. Activated Rho, Rac and Cdc42 were confirmed by performing respective activation G-LISA assays (RhoA, Rac1,2,3 and Cdc42 G-LISA Activation Assays; Cytoskeleton) with lysates from activated and control samples as per manufacturer's protocol. Overnight serum starved cells were treated as the control sample. To assess activity, samples were subsequently treated with 25 µg/ml calpeptin for 10 minutes or with 50 ng/ml EGF for 2 minutes. Activity of RhoA, Rac and Cdc42 respectively was assessed using a GLISA assay and normalized to values obtained for control lysates.

### Transient inhibition and activation of RhoGTPase

Specific inhibition and activation of the RhoGTPases was done by a method described by Burnham et al. [19] with minor modification. Plasmids expressing dominant negative Rac1 (pcDNA3-EGFP-Rac1-T17N), RhoA (pcDNA-EGFP-RhoA-T19N) and Cdc42 (pcDNA-EGFP-Cdc42-T17N), as well as plasmids expressing constitutively active mutants of RhoA (pcDNA-EGFP-RhoA-Q63L), Rac1 (pcDNA-EGFP-RhoA-Q61L) and Cdc42 (pcDNA-EGFP-Cdc42-Q61L) were kindly provided by Dr Garry Bokoch (The Scripps Research Institute, La Jolla, CA) [23], [52]. 5×10^4^ and 25×10^4^ cells were plated per well in a 24 and 6 well plate respectively, and cultured for 24 hours prior to transfection. For every 1 µg of DNA, 2 µl of Lipofectamine™ 2000 transfection reagent was added to the DNA solution, vortexed for 15 seconds and incubated at room temperature for 20 minutes. Complexes were added directly to the medium of the plated cells at a final DNA concentration of 1 µg per well for 24-well plates and 2.5 µg per well for 6-well plates. Analysis of GFP expression in transfected cells using FACS showed 30–40% cells to be successfully transfected with dominant negative or constitutively active genes and control plasmid (Fig. S2 A, B). 24 hours post transfection, cells were harvested and used to study the effect of RhoGTPase inhibition or activation on transgene expression as well as internalization. Activation or inhibition was analyzed by assessing RhoGTPases activity using respective GLISA assays (RhoA, Rac and Cdc42 G-LISA Activation Assays; Cytoskeleton) as per manufacturer's protocol. Cells transfected with pEGFP using Lipofectamine™ 2000, were used as control for studies involving transfection with dominant negative and constitutively active genes. For assessing activation, 24 hours post transfection with constitutive active genes, cells were serum starved overnight and then harvested for performing respective activation G-LISA assays and studying morphology. For assessing inhibition, 24 hours post transfection with dominant negative genes, cells were replated on fibronectin coated surface overnight and harvested for performing GLISA assays and studying morphology.

### Transfection

pEGFP-luc plasmid was purchased from clontech (Palo Alto, CA) and expanded using a Giga Prep Kit from Qiagen following the manufacturer's protocol. Linear poly-(ethylene imine) (25 kDa, PEI) was purchased from Polysciences (Warrington, PA). The cells were allowed to attach and cultured under various conditions as per required for the experiments mentioned. For gene transfer studies, DNA/PEI polyplexes were formed by mixing equal volumes of plasmid DNA with PEI. For every 1 µg of DNA, 1.65 µg of PEI was added to the DNA solution to get N/P of 12, vortexed for 15 seconds and incubated at room temperature for 15 minutes. Polyplexes were added directly to the medium of the plated cells at a final DNA concentration of 0.5 µg for 48-well plates. Salt was added directly to the wells post addition of transfection solution to get a final concentration of 150 mM NaCl. Transfection was quantified at 48 hours post transfection using the Promega Luciferase Assay System following the manufacturer's instructions. Subsequently, the total amount of protein in samples was analyzed using Peirce BCA assay with or without compat-Able protein assay preparation kit as per manufacturer's protocol. Experiments were carried out in triplicates and results were expressed as relative light units (RLU) per mg of cell protein.

### Internalization of polyplexes

Plasmid DNA (pEGFPluc) and the fluorescent DNA-intercalator YOYO-1 or YOYO-3 were mixed at a ratio of 1 dye molecule per 50 base pairs and allowed to complex for 60 minutes at room temperature. YOYO-1 or YOYO-3 labeled DNA was then used to prepare PEI/DNA complexes at a N/P of 12, bolus transfection was performed and cells were exposed to polyplexes for 2 hours. For YOYO-1 labeled complexes, cells were washed with PBS, trysinized with 50 µl of 0.25% trypsin-EDTA and suspended in 350 µl of 0.04% tryphan blue in 1% BGS in PBS to quench the flourescence of extracellular associated DNA. For YOYO-3 labeled complexes, cells were washed twice for 10 minutes with cellscrub to remove extracellular associated DNA, trysinized and suspended in 1% BGS in PBS. YOYO-1 fluorescent cells having were detected by flow cytometry with a FACScan X and data was analyzed with CELLQuest (Beckton Dickinson). YOYO-3 fluorescent cells were detected by flow cytometry using BD LSR II and data was analyzed with FACSDiVa (Beckton Dickinson). Experiments were performed in triplicates analyzing 7000 or 10000 total events per sample.

### Microscopy and quantification of stress fibers

D1 cells were plated on fibronectin coated plastic coverslips prior to culture. Immediately after treatment with Rho protein activators or inhibitors, cells were fixed with 5% paraformaldehyde for 15 minutes and permeabilized with 0.1% tritonX100 in 1× PBS for 3 minutes. The cells were stained for actin using Alexa488-phalloidin and for DNA using Hoechst 33258 dye. The staining solution was added in each well and left in dark for 30–60 minutes at room temperature followed by washings with 0.05% tween-20. Images were taken with a Zeiss AxioObserver Z1 inverted microscope at 100× magnification. The number of stress fibers per cell was quantified by counting the individual fibers observed in 100× pictures taken of cells in the respective condition.

### Statistical methods

All statistical analyses were performed using the computer program Instat (GraphPad, San Diego, CA). Experiments were statistically analyzed using a one-way ANOVA followed by a post-hoc test if the ANOVA result was p<0.05. Experiments were statistically analyzed using the Tukey test, which compares all pairs of columns, using a 95% confidence interval or using the Dunnet test which compares all columns versus a control column or using the unpaired t-test (two tail p value), which compares two different columns. Statistical significance was determined using a 95% confidence interval. Data is plotted as the mean of at least three independent measurements and standard deviation.

## Supporting Information

Figure S1 **Effect of inhibitors and activators on cell viability.** To study the effect of RhoGTPase inhibition on gene transfer, mMSCs were plated on fibronectin (40 µg/mL) coated tissue culture plastic surfaces for 16 hours prior to being treated with 0–300 ng/ml TcdB for 4 hours in serum free media, or 0–1 µg/ml C3 transferase for 4 hours. Cells were transfected immediately post treatment with inhibitors. For studying the effect of RhoGTPase activation, mMSCs were cultured for 8 hours on fibronectin, followed by overnight serum starvation and then treated with 0–100 µg/ml CP for 10 minutes or 0–100 ng/ml EGF for 2 minutes in serum free media. Subsequently, immediately post treatment with activators, bolus transfection was done for 4 hours in serum free media. For assessing the role of ROCK and PKC in gene transfer, mMSCs were cultured for 16 hours on fibronectin prior to treatment with 10 µM Y27632 (Y2) for 2 hours or 100 nM Staurosporine (ST) for 2 hours to inhibit ROCK and PKC, respectively. Immediately after treatment with ST or Y2, the medium was replaced and bolus transfection was done. Cell proliferation was analyzed immediately after treatment with TcdB (**A**), C3 transferase (**B**), CP (**C**), EGF (**D**) and ST or Y2 (**E**), as well 48 hrs post treatment and transfection by measuring calcien AM fluorescence after live/dead staining. The cell viability obtained after specific inhibitor treatment were statistically compared using Tukey-Kramer Multiple Comparison test, which compares all pairs with each other. The symbols *, **, and *** represents a significant change to the level of p<0.05, p<0.01, and p<0.001 respectively.
(TIF)

Figure S2 **Percent cells transfected with dominant negative and constitutively active genes, and the effect on cell viability.** To study the effect of direct inhibition and activation of RhoGTPase on gene transfer in cells plated on fibronectin tissue culture plastic, D1 cells were transiently transfected using lipofectamine™2000, with dominant negative or constitutively active forms of RhoA, Rac1 or Cdc42 conjugated with GFP. The distribution and percentage of cells transfected with dominant negative genes (**A**) or constitutively active genes (**B**), was assessed by analyzing GFP expression using flowcytometry. The cells were subsequently cultured on Fn for 16 hours prior to bolus transfection using linear polyethyleneimine (LPEI). The cell viability was determined 16 hours after culturing cells on fibronectin as well as 48 hours post addition of polyplexes using live/dead assay, for cells transfected with dominant negative genes (**C**) or constitutively active genes (**D**).
(TIF)
