# Supplementary figures and images for: Cellular Cytoskeleton Dynamics Modulates Non-Viral Gene Delivery through RhoGTPases
Source: PLoS One. 2012 Apr 11;7(4):e35046. doi: 10.1371/journal.pone.0035046 (PMC3324413; doi:10.1371/journal.pone.0035046)

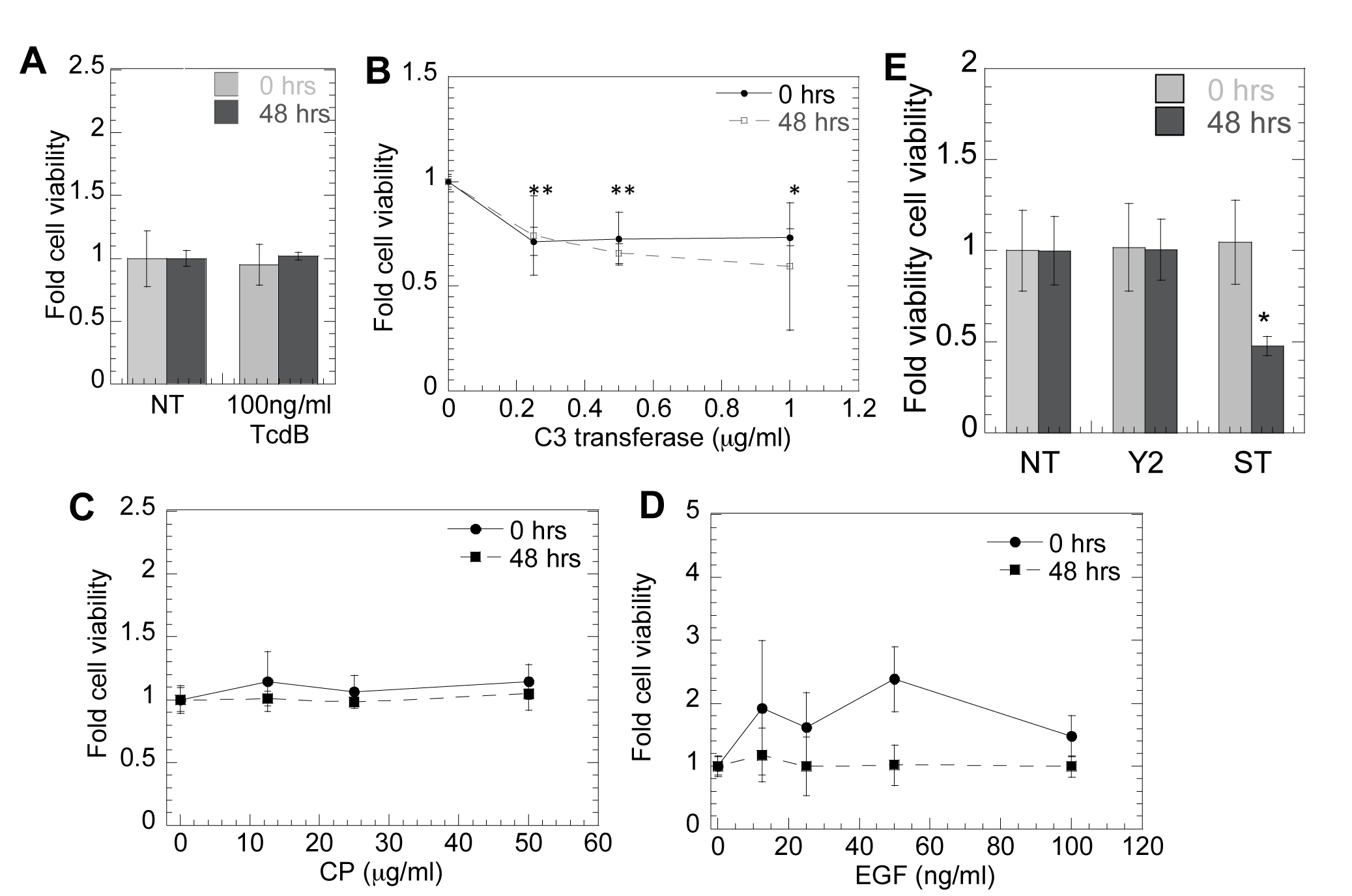

Supplement: Figure S1 — Effect of inhibitors and activators on cell viability. To study the effect of RhoGTPase inhibition on gene transfer, mMSCs were plated on fibronectin (40 µg/mL) coated tissue culture plastic surfaces for 16 hours prior to being treated with 0–300 ng/ml TcdB for 4 hours in serum free media, or 0–1 µg/ml C3 transferase for 4 hours. Cells were transfected immediately post treatment with inhibitors. For studying the effect of RhoGTPase activation, mMSCs were cultured for 8 hours on fibronectin, followed by overnight serum starvation and then treated with 0–100 µg/ml CP for 10 minutes or 0–100 ng/ml EGF for 2 minutes in serum free media. Subsequently, immediately post treatment with activators, bolus transfection was done for 4 hours in serum free media. For assessing the role of ROCK and PKC in gene transfer, mMSCs were cultured for 16 hours on fibronectin prior to treatment with 10 µM Y27632 (Y2) for 2 hours or 100 nM Staurosporine (ST) for 2 hours to inhibit ROCK and PKC, respectively. Immediately after treatment with ST or Y2, the medium was replaced and bolus transfection was done. Cell proliferation was analyzed immediately after treatment with TcdB (A), C3 transferase (B), CP (C), EGF (D) and ST or Y2 (E), as well 48 hrs post treatment and transfection by measuring calcien AM fluorescence after live/dead staining. The cell viability obtained after specific inhibitor treatment were statistically compared using Tukey-Kramer Multiple Comparison test, which compares all pairs with each other. The symbols *, **, and *** represents a significant change to the level of p<0.05, p<0.01, and p<0.001 respectively. (TIF) [file pone.0035046.s001.tif]

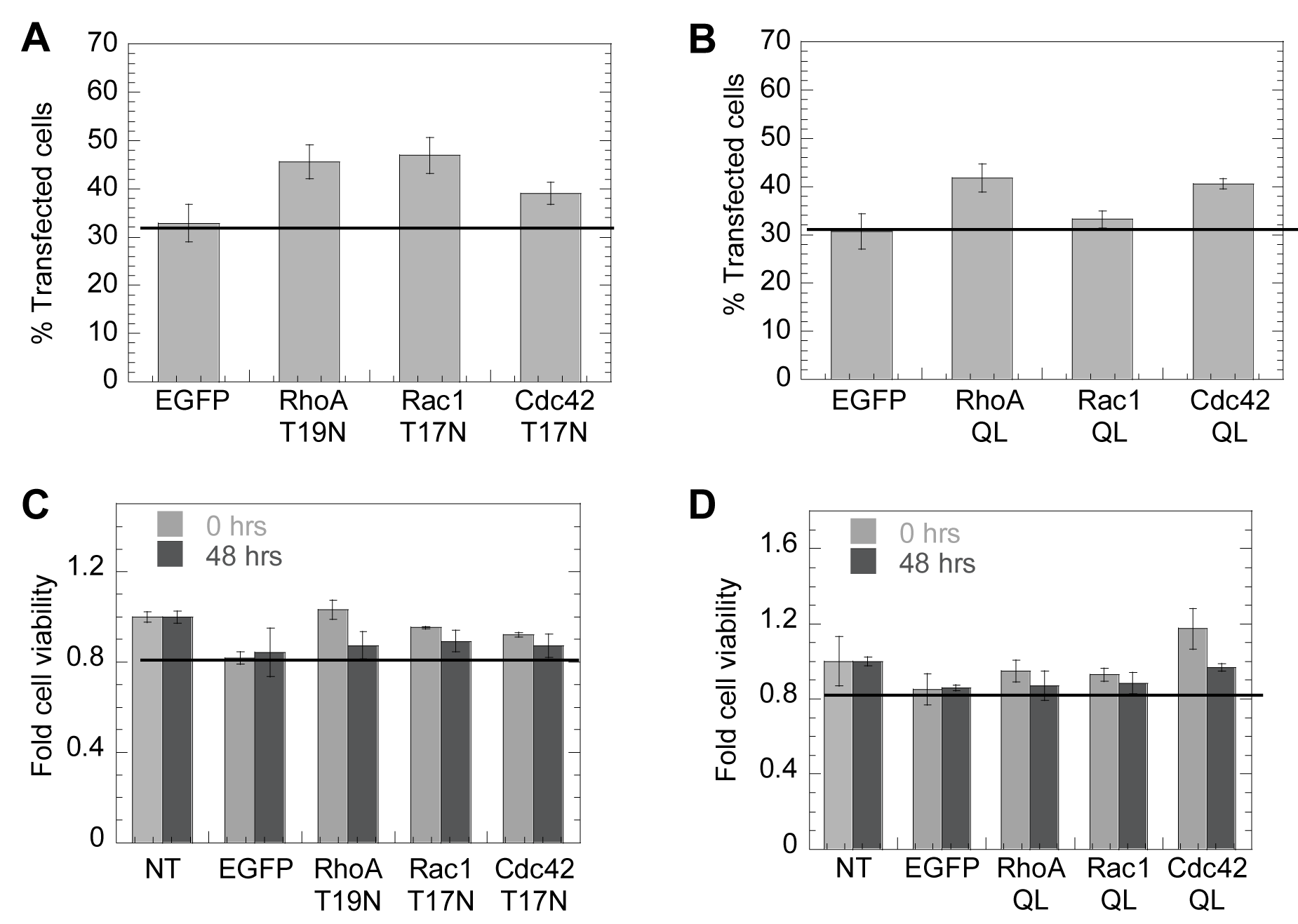

Supplement: Figure S2 — Percent cells transfected with dominant negative and constitutively active genes, and the effect on cell viability. To study the effect of direct inhibition and activation of RhoGTPase on gene transfer in cells plated on fibronectin tissue culture plastic, D1 cells were transiently transfected using lipofectamine™2000, with dominant negative or constitutively active forms of RhoA, Rac1 or Cdc42 conjugated with GFP. The distribution and percentage of cells transfected with dominant negative genes (A) or constitutively active genes (B), was assessed by analyzing GFP expression using flowcytometry. The cells were subsequently cultured on Fn for 16 hours prior to bolus transfection using linear polyethyleneimine (LPEI). The cell viability was determined 16 hours after culturing cells on fibronectin as well as 48 hours post addition of polyplexes using live/dead assay, for cells transfected with dominant negative genes (C) or constitutively active genes (D). (TIF) [file pone.0035046.s002.tif]
